# A new dual-scale nearest neighbor statistical feature construction algorithm for imbalanced data oriented to Gaussian naive bayes classifiers

**DOI:** 10.1038/s41598-026-57434-0

**Published:** 2026-06-13

**Authors:** Wei Wang, Shuang Ouyang, Fen Liu, Yanxi Li, Juan Pang

**Affiliations:** 1 Business School, Guilin Tourism University, Guilin, 541006 China; 2 Business School, University of International Business and Economics, Beijing, 100029 China; 3 Yulin Normal University, Yulin, 537000 China

**Keywords:** Imbalanced datasets, Gaussian Naive Bayes classifier, Dynamic dual-scale nearest neighbor statistical ratio, Feature construction algorithm, Engineering, Mathematics and computing

## Abstract

To address the performance degradation of Gaussian Naive Bayes (GNB) classifier on imbalanced datasets caused by sparse minority class features and severe class overlap, this paper proposes a new feature construction algorithm based on dynamic dual-scale nearest neighbor statistical ratio (NNDSR). The core of NNDSR is a dynamic dual-scale nearest neighbor mechanism, which is designed to accurately extract the local aggregation characteristics of samples and the inter-class boundary information. On this basis, new features are generated through cross-class and dual-scale statistical ratio operations. These features possess both strong discriminability and Gaussian distribution adaptability, which can significantly amplify class differences and effectively approximate the core assumptions of GNB. By optimizing the information expression of minority classes and enhancing class separability with these features, the algorithm avoids the information distortion problem of traditional sampling techniques and solves the mismatch between general feature enhancement algorithms and GNB’s core assumptions. Comparative experiments were conducted on 22 UCI datasets with varying scales, dimensions and imbalance ratios. Results show that NNDSR significantly outperforms the original data and 16 mainstream algorithms including sampling, feature enhancement and classifier-level optimization methods in core classification metrics such as AUC, G-mean and F-measure, with a notable improvement in the recognition accuracy of minority classes. Scalability tests further confirm its efficiency and stability on datasets with ten-thousand-level samples and within one hundred dimensions. This paper provides a robust new feature construction algorithm for GNB to handle imbalanced data, with strong practical application value.

## Introduction

In the era of big data, imbalanced data classification has become a core challenge in fields such as financial fraud detection, industrial equipment fault diagnosis, and medical rare disease screening. In such scenarios, minority-class samples are scarce but carry critical decision-making value, and their recognition accuracy often directly determines the practical effectiveness of the corresponding tasks.

Gaussian Naive Bayes (GNB) is widely adopted due to its high computational efficiency and strong interpretability. However, its performance often degrades notably on imbalanced data, which can be attributed to two key factors. On the one hand, the scarcity of minority-class samples may lead to biased estimation of the Gaussian distribution parameters (mean and variance) of the features. On the other hand, severe class overlap in the original feature space may be inconsistent with the core assumption of GNB that requires satisfactory feature separability. These characteristics make GNB a reasonable testbed for verifying imbalance learning algorithms. Improving the performance of GNB can directly reflect the effectiveness of methods in repairing distribution assumptions and enhancing feature discriminability.

Existing solutions still have difficulty in effectively overcoming the above obstacles. Traditional sampling methods, represented by SMOTE, can generate synthetic samples to balance the data distribution, but may easily bring about information distortion and overfitting. General feature enhancement algorithms, such as metric learning, can improve separability, but often disturb the Gaussian distribution and lead to mismatches with the inherent requirements of GNB. To alleviate such dilemmas, this paper proposes the Nearest Neighbor Distance Statistical Ratio (NNDSR) feature construction algorithm, which is tailored for GNB to relieve distribution bias and class overlap while maintaining Gaussian characteristics.

The core of NNDSR is a dynamic dual-scale nearest neighbor statistical framework. It adaptively divides a fine scale that focuses on the internal correlation of minority classes and a coarse scale that characterizes the boundary relationship with majority classes to extract local structural information. On this basis, new features are generated through dual-scale statistical ratio operations to quantify class differences into discriminative information. This design not only helps avoid the information distortion caused by sampling strategies but also mitigates the mismatch between general feature enhancement and GNB assumptions, enabling features to better approximate Gaussian distribution and highlight the differences of minority classes.

The innovations of this paper are threefold:

First, a dynamic dual-scale nearest neighbor statistical framework is proposed. Different from traditional algorithms relying on a fixed *k*-value neighborhood, NNDSR adaptively adjusts the neighborhood range through proportional parameters *α* and *β* ($\alpha <\beta$), and calculates the fine scale $k_{1}=\lfloor \alpha \cdot n_{1}\rfloor$ and coarse scale $k_{2}=\lfloor \beta \cdot n_{1}\rfloor$ according to the size of minority classes $n_{1}$. In this way, the shortcomings of fixed-scale methods can be relieved, including the noise sensitivity of small scales and the redundancy brought by large scales. The dual-scale collaboration captures the internal dense characteristics of minority classes and the inter-class boundary relationships, so as to accurately quantify the structural differences in imbalanced data.

Second, a statistical ratio feature construction mechanism is designed. Instead of global transformation or synthetic sample generation, NNDSR calculates six types of statistics and converts the abstract local class attribution tendency into concrete numerical features through cross-class and dual-scale ratio operations. This naturally amplifies inter-class differences and enhances noise resistance through statistical smoothing, thereby alleviating the problems of sampling-induced noise and the instability of generative feature algorithms.

Third, deep adaptation with GNB is realized. Most existing feature enhancement methods tend to ignore the adaptation to the assumptions of the target classifier. Based on the mathematical properties of distance statistical ratios, NNDSR ensures that new features better meet the core requirements of GNB: (1) distance statistics transformed by ratio operations are closer to Gaussian distribution; (2) feature dimensions are constructed based on relatively independent statistics, which helps satisfy conditional independence. Such a model-tailored feature construction alleviates the mismatch bottleneck between general feature engineering and specific models, and provides a feasible way to improve the performance of GNB on imbalanced data.

Comparative experiments show that NNDSR can significantly improve the classification performance of GNB on various imbalanced datasets, especially the recognition accuracy of minority classes.

The rest of this paper is organized as follows. “Related work” section reviews related work. “Method and technology” section details the core principles and implementation steps of NNDSR. “Comparative experiment design” section describes the experimental design and datasets. “Analysis of comparative experiment results” section analyzes the experimental results and comparisons. “Scalability test” section conducts scalability analysis. “Conclusion” section summarizes the work and discusses future directions.

## Related work

To improve the performance of GNB in imbalanced data scenarios, existing research mainly focuses on algorithm-level and data-level improvements. The two types of algorithms have their own emphases but both have unresolved limitations, providing room for improvement for this research.

### Algorithm-level improvements

Algorithm-level improvements focus on optimizing GNB’s own mechanism and reducing bias towards the majority class, mainly including three technical routes:

*Prior probability and weight optimization*: Enhance the model’s attention to the minority class by adjusting the prior probability estimation logic or assigning differentiated weights to samples, such as introducing distribution adaptive factors to optimize prior calculation^1^, or improving probability estimation through instance association structure^2,3^. However, such algorithms rely on manual parameter tuning and are difficult to improve the recognition accuracy of minority classes when class overlap is severe.

*Feature selection and weight adjustment*: Screen features closely related to the minority class or adjust feature weights to reduce redundant interference^4^; some studies also guide the model to focus on key information through feature weighting^5^. But this type of algorithm can only screen existing features and cannot generate new discriminative information, making it difficult to break through the separability bottleneck of the original feature space.

*Ensemble learning framework*: Combine multiple GNB classifiers to improve robustness^6^, or build a dedicated sub-classifier for the minority class. However, the ensemble design is complex and resource-consuming, and the diversity of base classifiers is difficult to control, which is prone to overfitting when the number of minority class samples is extremely small.

### Data-level improvements

Due to direct operation and easy implementation, data-level improvements have become the mainstream means to improve GNB’s performance, mainly divided into two technical routes: quantity adjustment and feature enhancement.

#### Quantity adjustment paradigm

The core of quantity adjustment is to alleviate the class imbalance problem by changing the number of samples, including three algorithms: over-sampling, under-sampling, and hybrid sampling:

***Over-sampling***: Increase the number of minority class samples by replicating minority class samples or generating new minority class samples. For example, the SMOTE algorithm generates minority class samples through interpolation, effectively alleviating the problem of insufficient minority class samples^7^; however, it is prone to introducing noise samples, especially in the boundary area of minority classes, which may lead to overfitting of the model to the minority class, and the generated samples are still based on the original feature space, failing to address the core challenge of the mismatch between features and GNB assumptions.

***Under-sampling***: Reduce the proportion of majority class samples by deleting redundant majority class samples. For example, some algorithms select core majority class samples based on nearest neighbor density and delete redundant samples overlapping with minority classes^8^; but under-sampling is prone to losing key information of the majority class, especially when the distribution of the majority class is uneven, which may destroy the overall structure of the data and lead to a decline in the generalization ability of the model.

***Hybrid sampling***: Combine the advantages of over-sampling and under-sampling, such as over-sampling the minority class first and then under-sampling the majority class^9^; however, such algorithms are essentially“surface quantity adjustment”, which do not touch on the problem of feature representation, and cannot solve the core problems of class overlap and Gaussian distribution parameter estimation deviation in the original feature space.

#### Feature enhancement paradigm

The core of feature enhancement is to optimize the original feature representation through feature transformation or generation, improving GNB’s adaptability to imbalanced data. In imbalanced data scenarios adapted to GNB, it mainly includes technologies such as metric learning^10^, kernel mapping^11^, and generative adversarial features^12^:

***Metric learning***: Optimize the class separability in the feature space by learning a new distance metric; however, existing metric learning is mostly designed for“globally balanced data”, without considering the characteristics of sparse minority class features and distribution shift in imbalanced data, making it difficult to learn effective metrics when the number of minority class samples is extremely small.

***Kernel mapping***: Convert original features to a high-dimensional space through non-linear mapping to improve class linear separability; but kernel mapping may make the distribution of new features deviate from the Gaussian distribution, conflicting with GNB’s core assumption of“features following Gaussian distribution”, which in turn leads to a decline in model performance.

***Generative adversarial features***: Use Generative Adversarial Networks (GAN) to generate new features conforming to the minority class distribution; although such algorithms can enrich the feature expression of the minority class, the generation process is complex and uncertain, which is prone to destroying the statistical stability of features and difficult to cooperate with GNB’s probabilistic modeling logic.

In addition, existing quantity adjustment and feature enhancement algorithms also have a common limitation of“single-scale dependence”: most algorithms rely on nearest neighbor information with a fixed *k* value to mine local structures, which cannot adapt to complex data distributions. When the scale is too small, only very local information can be captured, which is susceptible to noise interference, and may lead to feature distortion in sparse regions of the minority class. When the scale is too large, excessive redundant information of the majority class will be mixed in, blurring the boundary features of the minority class. This limitation makes it impossible to accurately mine the differentiated structures of“local dense areas of minority classes”and “surrounded areas of majority classes”in imbalanced data, and difficult to generate features that both conform to GNB assumptions and have strong discriminability.

Recently, a series of state-of-the-art methods have been proposed to address imbalanced classification from the oversampling perspective. These works mainly focus on handling class overlap, intra-class imbalance, feature noise, and complex minority-class structures by using density or closeness weighting, adaptive clustering, entropy-guided or cooling law-driven overlap cleaning, and weighted sample generation strategies. Although these methods significantly advance the capability of oversampling algorithms in handling complex data characteristics, they still predominantly rely on single-scale neighborhood modeling and fixed sampling regions or strategies. More importantly, they lack explicit consideration for the Gaussian distribution assumption required by GNB on conventional tabular imbalanced data, and do not incorporate a dual-scale statistical optimization mechanism to adapt to complex local distribution differences^13–17^.

### Common limitations of existing data-level improvement algorithms

In summary, existing data-level improvement algorithms, whether quantity adjustment or feature enhancement, have two unresolved common limitations when adapting to GNB to handle imbalanced data:

*Information distortion and assumption conflict*: Sampling algorithms change the data distribution, which may lose majority class information or introduce noise, destroying the parameter estimation basis of GNB; feature enhancement algorithms do not change the number of samples, avoiding the information loss or noise introduction problems of sampling techniques, but their improvement ideas often have fundamental assumption conflicts with GNB’s core premises of“features following Gaussian distribution”and“feature conditional independence”. For example, metric learning algorithms such as Large Margin Nearest Neighbor Metric Learning (LMNN)^10^ aim to learn a Mahalanobis distance metric, projecting data to a new feature space through linear transformation to increase the inter-class margin. However, this transformation optimized for“global linear separability”is likely to distort the original distribution of features, making the new features no longer follow the Gaussian distribution. Algorithms based on adversarial learning such as Adversarial Rebalancing Feature Learning (ARFL)^12^ may focus on the complexity of patterns rather than the regularity of distribution in the generated features. Similarly, kernel mapping algorithms map data to a high-dimensional space through non-linear transformation, which may improve linear separability, but the distribution form of the transformed features is difficult to control and often deviates from the Gaussian assumption. When these“non-Gaussian”features are input into GNB, the classifier makes decisions based on incorrect maximum likelihood estimation, and the reliability of its probability output is greatly reduced. This may instead become a bottleneck restricting its performance, resulting in difficulty in improving classification accuracy even if feature separability is improved, or even a decline. This is the deep-seated reason why many general feature enhancement algorithms are ineffective when paired with GNB.

*Single-scale dependence*: Both sampling algorithms such as SMOTE, along with its variants with fixed *k=5* nearest neighbors and the aforementioned feature enhancement algorithms mostly rely on a single, preset scale parameter such as the number of nearest neighbors *k* to extract local or global structural information. This static setting is difficult to adapt to complex local patterns in imbalanced data. When the scale is too small: classification is susceptible to noise samples, and in regions where the minority class is sparsely distributed, it is difficult to capture representative local structures, leading to unstable features. When the scale is too large: a lot of redundant information of the majority class is introduced, blurring class boundaries and diluting the discriminative information of the minority class that should be focused on. This“single-scale dependence”makes existing algorithms unable to accurately characterize the dual-scale structural differences from the“core area of the minority class”to the “inter-class boundary area”.

The innovation of the NNDSR proposed in this paper lies in directly providing solutions to the above two limitations:

For limitation 1 (assumption conflict): NNDSR abandons the complex global feature transformation idea and takes the lead in proposing a “statistical ratio”feature construction mechanism. New features are derived from distance calculations, and both the numerator and denominator are distance statistics. According to the central limit theorem, these statistics themselves are easy to asymptotically approximate the Gaussian distribution; furthermore, under most circumstances that satisfy classical statistical hypotheses, the ratio operation can also approximately preserve Gaussian characteristics (see “Dual-scale nearest neighbor statistic calculation” section for further details). Therefore, NNDSR is committed to being compatible with GNB’s Gaussian assumption from the mechanism of feature generation, fundamentally solving the“assumption conflict”problem.

For limitation 2 (single-scale dependence): NNDSR designs a dynamic dual-scale nearest neighbor mechanism, dynamically determining the “fine scale”($k_{1}$) and“coarse scale”($k_{2}$) according to the minority class sample size through proportional parameters *α* and *β*, focusing on core local and boundary information respectively. This dual-scale design can adaptively and comprehensively quantify the local class attribution tendency of samples, breaking through the limitations of a single fixed scale.

## Method and technology

### Basic idea of NNDSR

The idea of the NNDSR can be summarized as: first, mine the “affinity relationship”between samples and minority classes/majority classes in the local space; then quantify this relationship into new features with strong discriminability, amplifying the distribution differences between the two types of samples in the feature space; finally, accurately adapt to GNB’s dual core dependencies of“feature separability”and“Gaussian distribution assumption”.

Specifically, in imbalanced data, minority class samples usually form sparse local clusters, while majority class samples form dense global distributions. For any sample, there are significant differences between the distance statistical characteristics of its nearest neighbors with the minority class and those with the majority class. Generally, minority class samples are closer to their own class neighbors and farther from majority class neighbors, while majority class samples are the opposite. Therefore, by calculating the ratio of these two types of statistics, this“local attribution tendency”can be converted into numerical features, making the distribution differences between minority and majority classes more significant in the new feature space, thereby improving GNB’s ability to distinguish between the two types of samples.

### Overall framework overview

**Fig. 1:**
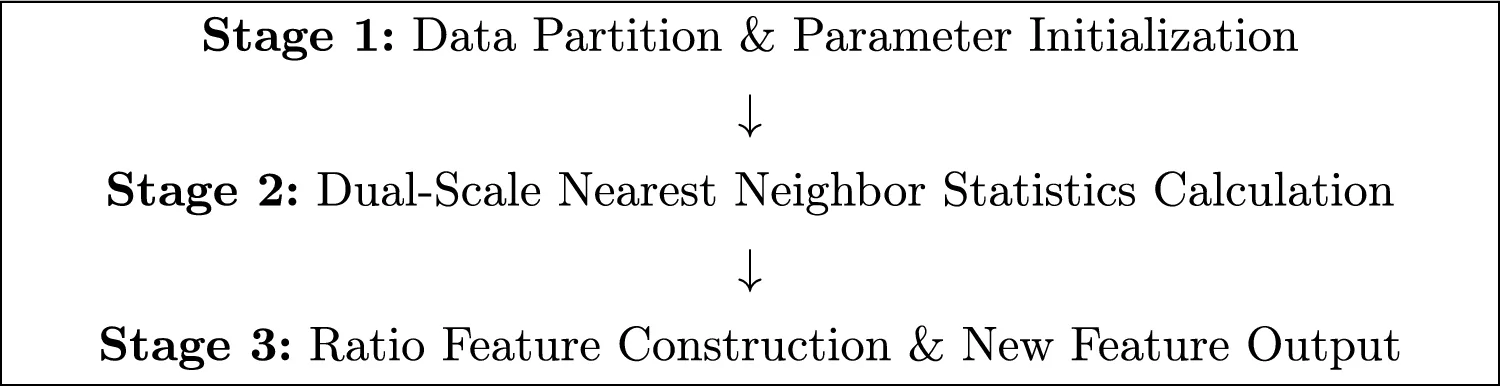
Overall framework of the proposed NNDSR algorithm.

The overall pipeline of the proposed NNDSR algorithm consists of three sequential stages, as illustrated in Fig. 1.

### Data symbol definition

Let the original imbalanced dataset be $D=\{(x_{i}, y_{i})\}_{i=1}^{N}$, where: - $x_{i} \in \mathbb {R}^{d}$ is a *d*-dimensional original feature vector; - $y_{i} \in \{0,1\}$ is the class label, $y_{i}=1$ represents the minority class, $y_{i}=0$ represents the majority class; - Minority class sample set $C_{1}=\{x_{i} \mid y_{i}=1\}$, sample size $n_{1}=|C_{1}|$; - Majority class sample set $C_{0}=\{x_{i} \mid y_{i}=0\}$, sample size $n_{0}=|C_{0}|$, $n_{1}+n_{0}=N$ and satisfying $n_{1} \ll n_{0}$.

### Dual-scale nearest neighbor statistic calculation

For each sample *x* in the dataset, calculate the distance statistics of its nearest neighbors with the minority class and majority class respectively. The number of nearest neighbors is dynamically adjusted based on the minority class sample size to avoid the adaptability problem of fixed nearest neighbors on datasets of different scales.

Let the proportional parameters $\alpha , \beta \in [0.1,0.9]$ and satisfy $\alpha <\beta$ to ensure the distinguishability of the two nearest neighbor scales. *α* and *β* can be adjusted according to the dataset characteristics. Then: - Number of nearest neighbors $k_{1}=\max (1,\left\lfloor \alpha \cdot n_{1}\right\rfloor )$; - Number of nearest neighbors $k_{2}=\max (1,\left\lfloor \beta \cdot n_{1}\right\rfloor )$.

Two nearest neighbor values $k_{1}$ and $k_{2}$ are selected, and their proportions are adjustable within the interval [0.1, 0.9]. The core reason is that they can capture the local class correlation of samples at dual scales: $k_{1}$ corresponds to the“closer neighborhood”, and $k_{2}$ corresponds to the“farther neighborhood”. A single nearest neighbor value can only characterize local information of a fixed scale, which is prone to reducing feature discriminability due to too narrow a neighborhood losing slightly distant correlations or too wide a neighborhood introducing redundant noise. Selecting three or more nearest neighbor values will lead to information redundancy in that statistics of different scales are highly correlated, and increase computational complexity in that the cost of nearest neighbor search and statistic calculation increases linearly with the number of nearest neighbors, resulting in diminishing marginal benefits.

The proportional parameters *α* and *β* are constrained within the range [0.1, 0.9] (instead of fixed values) with $\alpha < \beta$, mainly based on two key considerations to ensure reasonable neighborhood division: First, to avoid extreme proportions. A lower bound of 0.1 prevents extremely small neighbor numbers, which are sensitive to noise and local outliers; an upper bound of 0.9 avoids excessive neighbor selection, which would introduce numerous weakly correlated samples and weaken local distribution characterization. Second, to adapt to dataset differences. Different imbalanced datasets have distinct minority class distribution characteristics (e.g., dense or sparse). By adjusting *α* and *β*, the nearest neighbor scale can be flexibly optimized: for sparse datasets, the proportion can be increased to cover more valid neighbors; for dense datasets, the proportion can be reduced to focus on the core neighborhood. This design enhances the algorithm’s adaptability to various sample distributions while the constraint $\alpha < \beta$ clearly distinguishes fine-scale and coarse-scale neighborhoods, enabling the model to capture both intra-class compactness and inter-class boundary information.

The two typical parameter combinations adopted in experiments are selected according to the density and sparsity characteristics of different imbalanced datasets. Typically, *α =0.2* and *β =0.5* can be used by default, which balances the fineness and breadth of local information and serves as a benchmark configuration in most imbalanced scenarios. For further optimization, the suboptimal proportion combination can be searched through a grid algorithm.

- *Minority class nearest neighbor statistics*

For sample *x*, in the minority class subset $C_{1}=\{x_{i} \mid y_{i}=1\}$ of the training set, find $k_{1}$ nearest neighbors through the Euclidean distance $\left\| x-x'\right\| _{2}$, obtaining the distance set $D_{1, k_{1}}(x)=\{d_{(1)}, d_{(2)}, \dots , d_{(k_{1})}\}$ sorted in ascending order of distance.

Mean distance, reflecting the average proximity between the sample and its minority neighbors:

1 $$\begin{aligned} A_{1}(x)=\frac{1}{k_{1}} \sum _{j=1}^{k_{1}} d_{(j)} \end{aligned}$$

Variance of distances, reflecting the dispersion of neighbor distances:

2 $$\begin{aligned} A_{2}(x)=\frac{1}{k_{1}} \sum _{j=1}^{k_{1}}\left( d_{(j)}-A_{1}(x)\right) ^{2} \end{aligned}$$

Maximum distance, reflecting the farthest valid neighbor in the local range:

3 $$\begin{aligned} A_{3}(x)=d_{\left( k_{1}\right) } \end{aligned}$$

Similarly, for the distance set $D_{1, k_{2}}(x)$ of $k_{2}$ minority class nearest neighbors:

4 $$\begin{aligned} & B_{1}(x)=\frac{1}{k_{2}} \sum _{j=1}^{k_{2}} d_{(j)} \end{aligned}$$

5 $$\begin{aligned} & B_{2}(x)=\frac{1}{k_{2}} \sum _{j=1}^{k_{2}}\left( d_{(j)}-B_{1}(x)\right) ^{2} \end{aligned}$$

6 $$\begin{aligned} & B_{3}(x)=d_{\left( k_{2}\right) } \end{aligned}$$

b. *Majority class nearest neighbor statistics*

For sample *x*, in the majority class subset $C_{0}=\{x_{i} \mid y_{i}=0\}$ of the training set, find $k_{1}$ nearest neighbors through the Euclidean distance, obtaining the distance set $D_{0, k_{1}}(x)=\{d_{(1)}, d_{(2)}, \dots , d_{(k_{1})}\}$.

Mean distance:

7 $$\begin{aligned} E_{1}(x)=\frac{1}{k_{1}} \sum _{j=1}^{k_{1}} d_{(j)} \end{aligned}$$

Variance:

8 $$\begin{aligned} E_{2}(x)=\frac{1}{k_{1}} \sum _{j=1}^{k_{1}}\left( d_{(j)}-E_{1}(x)\right) ^{2} \end{aligned}$$

Maximum value:

9 $$\begin{aligned} E_{3}(x)=d_{\left( k_{1}\right) } \end{aligned}$$

Similarly, for $k_{2}$ neighbors in the majority class:

10 $$\begin{aligned} & F_{1}(x)=\frac{1}{k_{2}} \sum _{j=1}^{k_{2}} d_{(j)} \end{aligned}$$

11 $$\begin{aligned} & F_{2}(x)=\frac{1}{k_{2}} \sum _{j=1}^{k_{2}}\left( d_{(j)}-F_{1}(x)\right) ^{2} \end{aligned}$$

12 $$\begin{aligned} & F_{3}(x)=d_{\left( k_{2}\right) } \end{aligned}$$

### Ratio-based new feature space construction

To convert the“local class attribution tendency”of samples into discriminative features usable for classification, new features are constructed through the ratio of minority class statistics to majority class statistics. For each sample *x*, a 6-dimensional new feature vector $Z(x) \in \mathbb {R}^{6}$ is generated:

13 $$\begin{aligned} Z(x)=\left[ \begin{array}{lll} z_{1}=\frac{A_{1}(x)}{E_{1}(x)}, & z_{2}=\frac{A_{2}(x)}{E_{2}(x)}, & z_{3}=\frac{A_{3}(x)}{E_{3}(x)}, \\ z_{4}=\frac{B_{1}(x)}{F_{1}(x)}, & z_{5}=\frac{B_{2}(x)}{F_{2}(x)}, & z_{6}=\frac{B_{3}(x)}{F_{3}(x)} \end{array}\right] \end{aligned}$$

Reasons for adopting ratio features: First, to amplify class differences. For minority class samples, $A_{1}$, $A_{2}$, $A_{3}$ are small as they represent close distances to own class neighbors, and $E_{1}$, $E_{2}$, $E_{3}$ are large as they represent far distances to majority class neighbors, so $z_{1}-z_{3}$ take large values; for majority class samples, the opposite is true, and $z_{1}-z_{3}$ take small values, so the differences between the two classes are significantly amplified;

Second, to normalize the scale. Both the numerator and denominator are distance statistics (consistent dimension), and the ratio can eliminate the dimension difference of original features, making the feature distribution more stable;

Third, to enhance noise resistance. The noise interference of individual nearest neighbors is smoothed through statistics such as mean and variance, enhancing the robustness of features.

Handling of special cases: If the denominator such as $E_{3}(x)$ is 0, in extreme cases, a very small value $10^{-6}$ can be used instead to avoid numerical calculation abnormalities.

**Algorithm 1:**
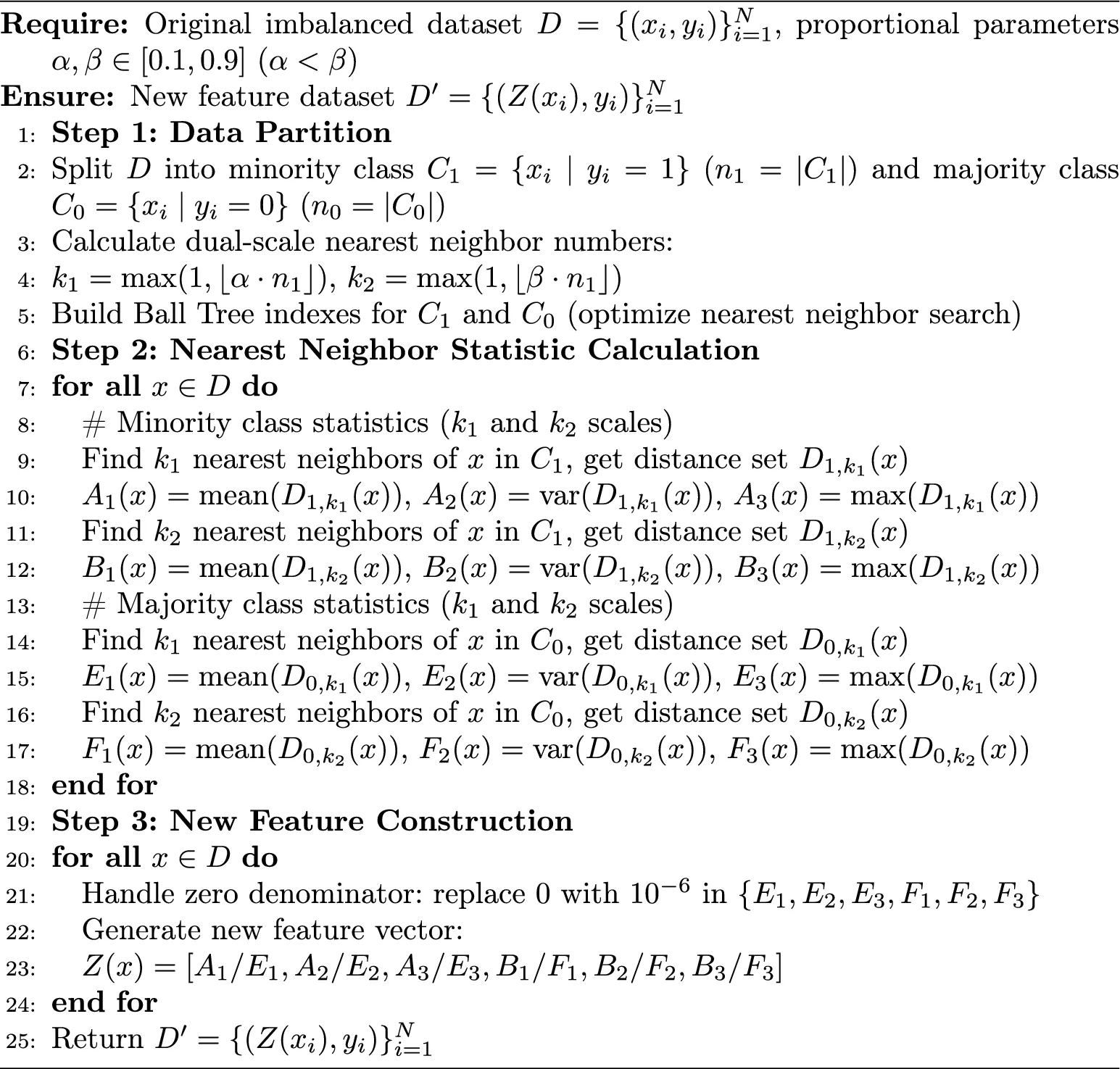
NNDSR.

### Intuitive explanation of algorithm design

#### “Microscope-telescope” model of dual-scale nearest neighbors

The role of the dual-scale nearest neighbor mechanism can be vividly understood as a“microscope-telescope”model: - $k_{1}$ (local microscope): Focuses on the core neighborhood to capture the close correlation between the sample and its own class. For example, when minority class samples are clustered, most of the $k_{1}$ nearest neighbors are of the same class, and $A_{1}$ is small, reflecting “cohesion”. - $k_{2}$ (boundary telescope): Extends to the edge neighborhood to capture the boundary relationship with heterogeneous classes. For example, the $k_{2}$ nearest neighbors of minority class samples at the boundary may include majority class samples, and $B_{1}$ is larger than $A_{1}$, reflecting“boundary crossing”.

#### Complementarity of three types of statistics

***Mean***: Reflects the average distance. The $A_{1}$ of minority class samples is usually smaller than that of majority class samples.

***Variance***: Reflects the dispersion degree of the distance distribution. The $E_{2}$ of majority class samples is often small, corresponding to a dense distribution, and the $A_{2}$ of minority class samples may be large, indicating large intra-cluster variation.

***Maximum value***: Reflects the boundary range. The $E_{3}$ of minority class samples is usually large, indicating a far distance from the majority class. The combination of the three can comprehensively describe the local distribution, avoiding the limitations of a single metric.

#### “Amplifier” effect of ratio features

The ratio of minority class statistics to majority class statistics can significantly expand inter-class differences. The $z_{1}=A_{1}(x) / E_{1}(x)$ of minority class samples tends to be smaller since $A_{1}$ is small, $E_{1}$ is large, and the $z_{1}=A_{1}(x) / E_{1}(x)$ of majority class samples tends to be larger since $A_{1}$ is large, $E_{1}$ is small. The distribution centers of $z_{1}$ of the two types of samples are significantly separated, making it easier for GNB to learn the decision boundary.

### Complexity analysis of NNDSR

In terms of time complexity, the core time consumption of the NNDSR is concentrated in Ball Tree construction and nearest neighbor query. The time complexity of Ball Tree construction for both minority class and majority class is *O(n łog n)*, which can be approximately *O(N łog N)* overall; each sample needs to perform 4 *k*-nearest neighbor queries, and the time complexity of a single query is *O(łog n + k)*. The total query time is *O(N łog N)*. Therefore, the overall time complexity is *O(N łog N)*.

In terms of space complexity, the NNDSR mainly relies on sample storage and intermediate statistics: the original samples need to store $n_{1}$ minority class samples and $n_{0}$ majority class samples, with space *O*(*Nd*); the space of Ball Tree index, statistic matrix, and new feature matrix are all *O*(*N*), and the overall space complexity is *O*(*Nd*), which is linearly related to the original data dimension and scale.

Overall, the computational complexity of the NNDSR is generally at a medium level, and its complexity is suitable for medium-scale high-dimensional imbalanced data. For large-scale scenarios, the efficiency of nearest neighbor search needs to be optimized.

Compared with typical baselines, SMOTE requires additional synthetic sample generation and multi-neighbor searching, which increases computational consumption under highly imbalanced tasks. LMNN depends on iterative metric learning optimization and parameter updating, resulting in heavy time cost. In contrast, NNDSR only performs dual-scale nearest neighbor statistics and simple ratio calculation, maintaining stable *O(Nłog N)* complexity and superior efficiency for large-scale and high-dimensional datasets.

## Comparative experiment design

### Experiment overview

This chapter aims to comprehensively evaluate the performance of the proposed NNDSR algorithm for imbalanced data classification, with Gaussian Naive Bayes (GNB) as the benchmark classifier. The core objectives are: (1) Verify the advantages of NNDSR in enhancing minority class recognition and improving overall GNB performance by comparing with original data and mainstream imbalanced data processing algorithms; (2) Analyze the robustness and generalization ability of NNDSR across datasets with varying sample sizes, imbalance ratios, and data types.

The selection of GNB as the benchmark is based on two key considerations: Academic comparability: GNB is a widely adopted benchmark in imbalanced data research, enabling direct comparison with existing studies and ensuring the practical reference value of results; Algorithm pertinence: NNDSR addresses the inherent limitations of traditional methods—avoiding information distortion from sampling algorithms and resolving the mismatch between general feature enhancement algorithms and GNB’s “Gaussian distribution assumption” and “conditional independence assumption”, thus highlighting its unique value for GNB optimization.

### Experimental setup

#### Hardware and software environment

All experiments are implemented in Python 3.10 on the Spyder platform, running on a 64-bit Windows 11 operating system. The hardware environment is equipped with an Intel Core i7 processor and 16 GB of memory.

#### Dataset construction

To ensure comprehensive scenario coverage, datasets are constructed following the principles of diversity, representativeness, and consistency.

***Dataset source*** 11 authoritative datasets are selected from the UCI Machine Learning Repository, covering biology, medicine, network security, and other fields (Ecoli^18^, Haberman^19^, Iris^20^, Glass^21^, Letter-recognition^22^, Page-blocks^23^, Pima^24^, Phishingdata^25^, Poker-hand^26^, Seeds^27^, Yeast^28^). The selection rationale includes: Authority: Standardized annotation and wide adoption in benchmarking ensure reproducibility; Scene representativeness: Corresponding to real-world imbalanced tasks (e.g., rare disease screening, fraud detection); Inherent diversity: Natural variations in sample size, attribute count, and data type without additional transformation.

***Imbalanced dataset construction rules*** Based on the original datasets, 22 binary imbalanced datasets are generated to simulate diverse real-world scenarios (see Table 1), with design rules as follows: Sample size and attribute diversity: Cover small-scale (*<1000* samples), medium-scale (*1000 łe* samples *<5000*), and large-scale (*\ge 5000* samples) datasets; low-to-medium dimensional (*<10*) and high-dimensional (*\ge 10* attributes) features. This verifies computational efficiency and high-dimensional adaptability. Imbalance ratio diversity: Include high imbalance (*>1:10*), medium imbalance (1 : 10–1 : 5), and low imbalance (1 : 5–1 : 2) to test statistical stability under extreme scarcity. Data type comprehensiveness: Cover continuous, discrete, and mixed-type data to evaluate robustness to feature forms. Non-binary conversion: Convert multi-class datasets to binary via “one-vs-one”, “one-vs-many”, or “many-vs-many” strategies to focus on the core “minority vs majority” contradiction.

**Table 1:** Experimental dataset information.

Dataset name	Attributes	Sample size	Class	Imbalance ratio	Data type
Ecoli	7	336	imU:others	1:8.6	Continuous data
Glass1	9	214	1:others	1:2.06	Continuous data
Glass2	9	214	6,7:others	1:4.63	Continuous data
Haberman	3	306	2:1	1:2.78	Mixed data
Iris	4	150	1:others	1:2	Continuous data
Letter-recognition1	16	20000	A,B:others	1:11.86	Discrete data
Letter-recognition2	16	20000	C,D,E,F:others	1:5.49	Discrete data
Letter-recognition3	16	20000	G:others	1:24.87	Discrete data
Page-blocks	10	5473	others:1	1:8.77	Mixed data
Pima	8	768	1:0	1:1.87	Mixed data
Poker-hand1	10	25010	others:1,0	1:12.04	Discrete data
Poker-hand2	10	25010	2:others	1:19.74	Discrete data
Phishingdata1	9	1353	0:others	1:12.14	Mixed data
Phishingdata2	9	651	0:1	1:5.32	Mixed data
Phishingdata3	9	805	0:-1	1:6.82	Mixed data
Seeds	7	210	1:others	1:2	Continuous data
Yeast1	8	1484	CYT:others	1:2.21	Mixed data
Yeast2	8	1484	MIT:others	1:5.08	Mixed data
Yeast3	8	1484	ME3:others	1:8.10	Mixed data
Yeast4	8	1484	ME2:others	1:28.10	Mixed data
Yeast5	8	1484	ME1:others	1:32.72	Mixed data
Yeast6	8	514	ME2:CYT	1:9.08	Mixed data

Dataset source: UCI Machine Learning Repository

#### Comparative algorithms

To ensure comprehensive evaluation, 17 mainstream algorithms are selected across five categories, covering “data-level improvement” and “classifier-level optimization” paths:

In the comparative experiments, we select 16 typical algorithms from five categories as baselines:

1. Feature enhancement methods: LMNN^10^, ARFL^12^, RP-KFE^11^;
2. Over-sampling methods: SMOTE^7^, MWMOTE^29^, K-Means SMOTE^30^, DPSMOTE^31^, SVMSMOTE^32^, LDBSMOTE^33^, IBSM^34^;
3. Hybrid sampling methods: SMOTETomek, SMOTEENN^9^;
4. Under-sampling methods: Tomek Links^8^, AllKNN^35^;
5. Classifier-level optimization methods: CBFW^5^, CBFW-G^5^.

Rationale for algorithm selection: Over-sampling algorithms are prioritized for their wide application and ability to retain original data information; Covering two technical paths (data processing vs. classifier optimization) enables accurate comparison of NNDSR’s advantages under unified GNB framework.

#### Experimental protocol

***Data preprocessing*** Missing value handling: Median filling for continuous features, mode filling for categorical features; remove features with *>30%* missing values. Categorical encoding: One-hot encoding for unordered features, label encoding for ordered features. No standardization/normalization: Avoid distorting original feature scales, which are critical for NNDSR’s Euclidean distance-based affinity measurement.

***Parameter optimization*** NNDSR: Grid search for $\alpha , \beta$ in [0.1, 0.9] (step size 0.1); Comparative algorithms: Parameter optimization within classic value ranges; GNB: Adaptive adjustment of prior probability based on class distribution.

***Sampling rules*** Over-sampling: Stop at 1:1 minority-majority ratio (follow original algorithm rules if specified); Under-sampling: Grid search for optimal ratio to retain key majority class information; Feature enhancement (including NNDSR): No sample count change, only feature transformation.

***Algorithm implementation*** Classic algorithms: Implemented via imblearn/scikit-learn libraries; Recent algorithms: Coded following original papers to ensure core logic consistency.

### Performance evaluation

#### Evaluation metrics

Three core metrics^36^ are selected to address imbalanced data characteristics:

AUC (Area Under ROC Curve): Measures overall discriminative ability, unaffected by classification thresholds. Defined as:

14 $$\begin{aligned} \text {AUC} = \mathbb {P}(P_+ > P_-) \end{aligned}$$

where $P_+$ and $P_-$ are predicted probabilities of positive (minority) and negative (majority) samples, respectively.

G-mean: Balances minority and majority class performance, calculated as the geometric mean of TPR (True Positive Rate) and TNR (True Negative Rate):

15 $$\begin{aligned} \text {G-mean} = \sqrt{\text {TPR} \times \text {TNR}} \end{aligned}$$

with:

16 $$\begin{aligned} \text {TPR} = \frac{TP}{TP + FN}, \quad \text {TNR} = \frac{TN}{TN + FP} \end{aligned}$$

where *TP* (True Positive), *FN* (False Negative), *TN* (True Negative), and *FP* (False Positive) are confusion matrix components.

F-measure: Focuses on minority class precision and recall (harmonic mean):

17 $$\begin{aligned} \text {F-measure} = \frac{2 \times \text {Precision} \times \text {Recall}}{\text {Precision} + \text {Recall}} \end{aligned}$$

with:

18 $$\begin{aligned} \text {Precision} = \frac{TP}{TP + FP}, \quad \text {Recall} = \text {TPR} \end{aligned}$$

#### Verification strategy

Stratified fivefold cross-validation: 80% training set, 20% test set, maintaining original class ratio in each fold; 10 independent repetitions: Redesign cross-validation folds and re-optimize parameters per repetition; Result presentation: Mean ± standard deviation to reflect algorithm stability.

## Analysis of comparative experiment results

To verify the effectiveness and adaptability of the NNDSR, this paper compares the performance of NNDSR with various algorithms on 22 imbalanced datasets through quantitative results and visualization analysis.

### Overall performance comparison

Comprehensive experimental results on 22 datasets show that the performance of the NNDSR is comprehensively superior to all comparative algorithms and the original data. The results of each algorithm in various performance evaluation metrics on all datasets are detailed in Appendix Tables 8, 9, 10, 11, 12 and 13.

The ranking rule for this time is: in each performance evaluation metric, the algorithm with the largest metric value is ranked first, and the other algorithms and original data are ranked from 2nd to 18th in descending order of metric values.

**Fig. 2:**
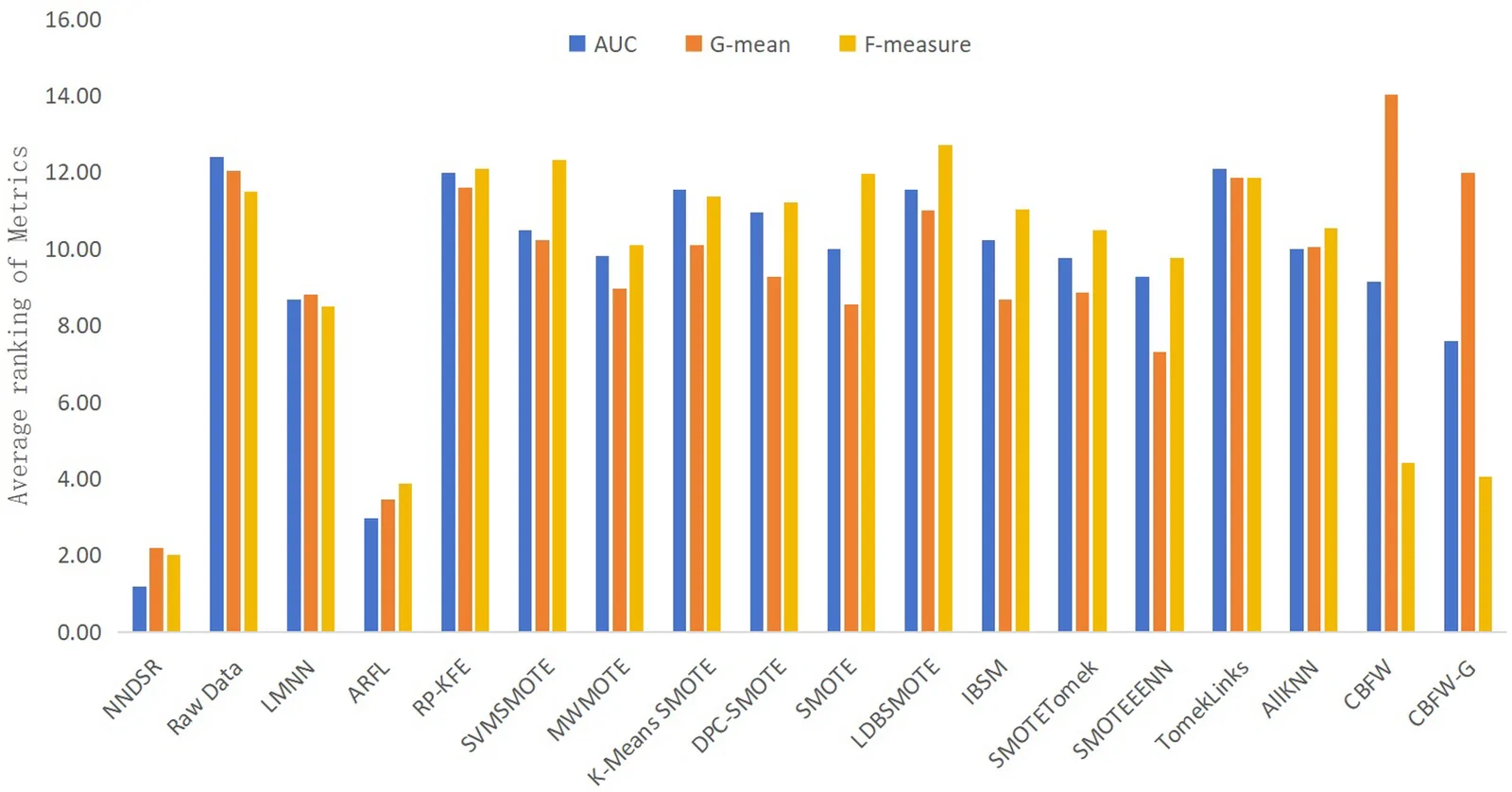
Average ranking of each algorithm on three metrics.

From the ranking performance, NNDSR ranks first in 20 datasets in the AUC metric, 14 datasets in the G-mean metric, and 15 datasets in the F-measure metric. NNDSR is the only algorithm that occupies the “optimal position in the vast majority of datasets”in all three metrics. The average ranking of NNDSR is also ahead of other comparative algorithms, with average rankings of 1.18, 2.18, and 2.00 in AUC, G-mean, and F-measure respectively. The average rankings of each algorithm in all performance evaluation metrics on all datasets are shown in Fig. 2.

Compared with the overall mean level of all comparative algorithms, NNDSR achieves significant improvements in the three core metrics. In the AUC metric, the combined mean value of all comparative algorithms is 0.6752, and NNDSR exceeds it by 30.45% with 0.8808; in the G-mean metric, the combined mean value of comparative algorithms is 0.5995, and NNDSR exceeds it by 37.53% with 0.8245; while in the F-measure metric focusing on minority class recognition accuracy, the improvement is particularly prominent. The combined mean value of comparative algorithms is 0.4386, and NNDSR exceeds it by 69.20% with 0.7421, fully reflecting its core advantage in enhancing the discriminability of minority class features.

This comprehensive advantage fully verifies the effectiveness and robustness of NNDSR in handling imbalanced data.

### Statistical significance test of comparative experiment results

To scientifically verify the effectiveness of the experimental results between the NNDSR and other algorithms, this paper adopts a rigorous statistical test process. First, the Friedman test^37^ is used to comprehensively evaluate the performance of all algorithms on different datasets. This non-parametric algorithm is suitable for comparing differences among multiple related samples. The test results are shown in Table 2. The *p*-values of all performance metrics under each classifier are less than 0.05, indicating that there are significant differences in algorithm performance.

**Table 2:** Friedman test results.

Metrics	AUC	G-mean	F-measure
*P* value	8.48e-50	4.91e-48	8.47e-50

After confirming the overall difference, this paper uses the Mann-Whitney U test^38^ for pairwise comparison. This non-parametric algorithm can accurately reveal the specific gap between NNDSR and other algorithms, and is suitable for non-normally distributed data. Considering that multiple tests may increase errors, the Bonferroni correction^39^ is used to adjust the significance level, effectively controlling Type I errors. The test and correction results are shown in Table 3. The *p*-values and corrected values of the comparison between NNDSR and other algorithms are all lower than the significance threshold of 0.05. In summary, the statistical results clearly show that NNDSR has significant improvements in classification metrics compared with the original data and 16 comparative algorithms.

**Table 3:** Mann-Whitney U test and Bonferroni correction results.

Algorithm	AUC		G-mean		F-measure	
	Corrected *P* value	*P* value	Corrected *P* value	*P* value	Corrected *P* value	*P* value
Raw Data	4.12e-08	2.43e-09	1.03e-06	6.04e-08	6.74e-07	3.96e-08
LMNN	1.43e-07	8.41e-09	8.36e-04	4.92e-05	4.44e-06	2.61e-07
ARFL	2.92e-06	1.72e-07	3.88e-02	2.28e-03	1.41e-03	8.28e-05
RP-KFE	4.25e-08	2.50e-09	1.14e-06	6.69e-08	7.25e-07	4.26e-08
SVMSMOTE	5.45e-08	3.21e-09	1.98e-05	1.16e-06	1.17e-06	6.89e-08
MWMOTE	5.41e-08	3.18e-09	5.33e-06	3.14e-07	1.42e-06	8.35e-08
KSMOTE	5.44e-08	3.20e-09	3.48e-06	2.04e-07	7.65e-07	4.50e-08
DPCSMOTE	4.97e-08	2.93e-09	5.03e-06	2.96e-07	1.23e-06	7.26e-08
SMOTE	4.21e-08	2.47e-09	6.74e-06	3.96e-07	1.38e-06	8.15e-08
LDBSMOTE	7.15e-08	4.21e-09	5.55e-06	3.27e-07	5.86e-07	3.44e-08
IBSM	5.02e-08	2.95e-09	5.72e-06	3.37e-07	1.49e-06	8.78e-08
SMOTETomek	4.94e-08	2.91e-09	4.38e-06	2.58e-07	2.68e-06	1.58e-07
SMOTEENN	5.81e-08	3.41e-09	6.17e-05	3.63e-06	1.43e-06	8.40e-08
TomekLinks	4.25e-08	2.50e-09	1.09e-06	6.40e-08	6.52e-07	3.84e-08
AllKNN	5.78e-08	3.40e-09	2.23e-05	1.31e-06	1.15e-06	6.74e-08
CBFW	6.17e-07	3.63e-08	5.19e-07	3.05e-08	9.55e-03	5.62e-04
CBFW-G	1.36e-07	7.99e-09	1.31e-06	7.69e-08	8.46e-04	4.98e-05

### Comparative analysis with different types of algorithms

The mean values of different types of algorithms in each classification performance metric are detailed in Table 4. By integrating the mean metric values of similar algorithms, this table intuitively presents the overall performance characteristics of different types of algorithms, providing a clear data reference for horizontal comparison of the comprehensive performance of various types of algorithms.

**Table 4:** Average performance metrics of different algorithms.

Metric	NNDSR	Feature enhancement	Over sampling	Under sampling	Hybrid sampling	Classifier optimization	Raw data
AUC	0.8808	0.7541	0.7014	0.7005	0.7140	0.7385	0.6924
G-mean	0.8245	0.6799	0.6520	0.5954	0.6699	0.5288	0.5814
F-measure	0.7421	0.5082	0.4200	0.4245	0.4312	0.6402	0.4246

Compared with feature enhancement algorithms, the mean values of the three core metrics of NNDSR are all significantly leading. From the perspective of the AUC metric, the mean value of feature enhancement algorithms is 0.7541, and NNDSR (0.8808) is 16.80% ahead of it, showing stronger overall classification discrimination ability. From the perspective of the G-mean metric, NNDSR (0.8245) is 21.27% higher than the mean value of feature enhancement algorithms (0.6799), showing obvious advantages in balancing the classification effect of positive and negative classes. From the perspective of the F-measure metric, NNDSR (0.7421) is 1.46 times the mean value of feature enhancement algorithms (0.5082), further highlighting its core advantage in minority class recognition accuracy.

Compared with over-sampling algorithms, the overall performance advantage of NNDSR is more prominent. The mean AUC value of over-sampling algorithms is 0.7014, which is only 79.63% of NNDSR (0.8808). Even the better-performing models among over-sampling algorithms are difficult to reach the AUC level of NNDSR; in terms of G-mean, the mean of over-sampling algorithms is 0.6520, which is 20.92% lower than NNDSR (0.8245). This is mainly because over-sampling synthetic samples are prone to introducing noise, leading to blurred class boundaries and affecting the balanced classification effect; in terms of F-measure, the mean of over-sampling algorithms is 0.4200, which is 56.59% lower than NNDSR (0.7421). Especially on highly imbalanced datasets, over-sampling is prone to causing over-fitting problems of minority classes, leading to a significant drop in F-measure.

Compared with under-sampling algorithms and hybrid sampling algorithms, the average performance of NNDSR also ranks first. In the AUC metric, the mean of hybrid sampling algorithms is 0.7140, and the mean of under-sampling algorithms is 0.7005, which are 18.94% and 20.47% lower than NNDSR (0.8808) respectively; in the G-mean metric, due to the easy loss of key information caused by deleting majority class samples in under-sampling, the mean values of the two types of algorithms are significantly lower than NNDSR (0.8245), which are 18.75% and 27.79% lower respectively; in the F-measure metric, the mean of hybrid sampling is 0.4312, and the mean of under-sampling is 0.4245, which are only 58.11% and 57.20% of NNDSR. The advantage of NNDSR in minority class recognition is further highlighted.

In addition, compared with classifier-level optimization algorithms and original data, the performance advantage of NNDSR further confirms the effectiveness of its improvement ideas. The mean AUC of classifier-level optimization algorithms is 0.7385, the mean G-mean is 0.5288, and the mean F-measure is 0.6402, which are 16.16%, 35.86%, and 13.73% lower than NNDSR respectively. Especially in G-mean, the gap is most obvious because the class distribution is not optimized from the data level; the performance of the original data in various metrics is the worst. Its AUC is only 78.61% of NNDSR, G-mean is less than 70.52% of NNDSR, and F-measure is only 57.22% of NNDSR. This fully shows that the design of NNDSR in data processing and feature optimization plays a key role in improving the classification performance of imbalanced data.

### Analysis by dataset characteristics

#### Low-to-medium dimensional small-scale datasets

A total of 16 low-to-medium dimensional small-scale datasets (dimension *łe 9*, sample size *łe 2000*) are selected, covering continuous data and mixed data. The core challenge of such data lies in class boundary overlap, which easily leads to model misjudgment. NNDSR breaks through this bottleneck by amplifying local class differences, and its performance is as follows:

In terms of the AUC metric:The mean value of NNDSR on this type of dataset reaches 0.8779, which is 17.81% higher than the combined mean value (0.7452) of all comparative algorithms. Even on datasets with severe overlap such as Haberman (AUC 0.6701), it still outperforms most comparative algorithms .

In terms of the G-mean metric: The mean value of NNDSR is 0.8263, which is 22.81% higher than the mean value (0.6728) of comparative algorithms, effectively balancing the classification effect of positive and negative classes. Taking Glass1 as an example, the G-mean of NNDSR (0.7879) is 16.99% and 15.46% higher than that of Raw Data (0.6735) and LMNN (0.6824) respectively, highlighting its ability to optimize overlapping boundaries.

In terms of the F-measure metric: The mean value of NNDSR reaches 0.7312, which is 48.77% higher than the mean value (0.4915) of comparative algorithms. Especially on datasets with a low proportion of minority classes, the F-measure of NNDSR is still much higher than that of Raw Data, confirming its effect on alleviating the class overlap problem.

#### High-dimensional large-scale datasets

A total of 5 high-dimensional large-scale datasets (dimension *\ge 10*, sample size *\ge 20000*) are selected, including discrete data. The mixed data Page-blocks is excluded because its sample size (5473) is less than 20000. Such data is prone to the“curse of dimensionality”due to high dimensions (10-16 dimensions), large sample sizes (20000-25010), and high imbalance ratios, and minority class features are easily“drowned”by a large amount of redundant information. NNDSR captures the local structure of high-dimensional space through dual-scale nearest neighbor statistics, and its performance improvement is more prominent:

In terms of the AUC metric: The mean value of NNDSR is 0.8603, which is 31.77% higher than the combined mean value (0.6529) of comparative algorithms; even compared with ARFL (mean 0.7727), the best-performing feature enhancement algorithm in this scenario, it is still 11.34% higher.

In terms of the G-mean metric: The mean value of NNDSR reaches 0.8015, which is 40.17% higher than the mean value (0.5718) of comparative algorithms; it is 12.98% higher than ARFL (mean 0.7094), effectively solving the problem of blurred class boundaries in high-dimensional data. For example, the G-mean of NNDSR (0.5560) on Poker-hand2 is 1.68 times that of ARFL (0.3311).

In terms of the F-measure metric: The mean value of NNDSR is 0.7424, which is 197.43% higher than the mean value (0.2496) of comparative algorithms; it is 70.55% higher than ARFL (mean 0.4353).The advantage is particularly obvious on extremely imbalanced datasets such as Poker-hand1 and Poker-hand2, where the F-measure of NNDSR reaches 0.5803 and 0.5026 while that of ARFL is only 0.1954 and 0.0861, fully proving that it can rescue minority class features “drowned”in high-dimensional discrete data.

### Verification by visualization results

**Fig. 3:**
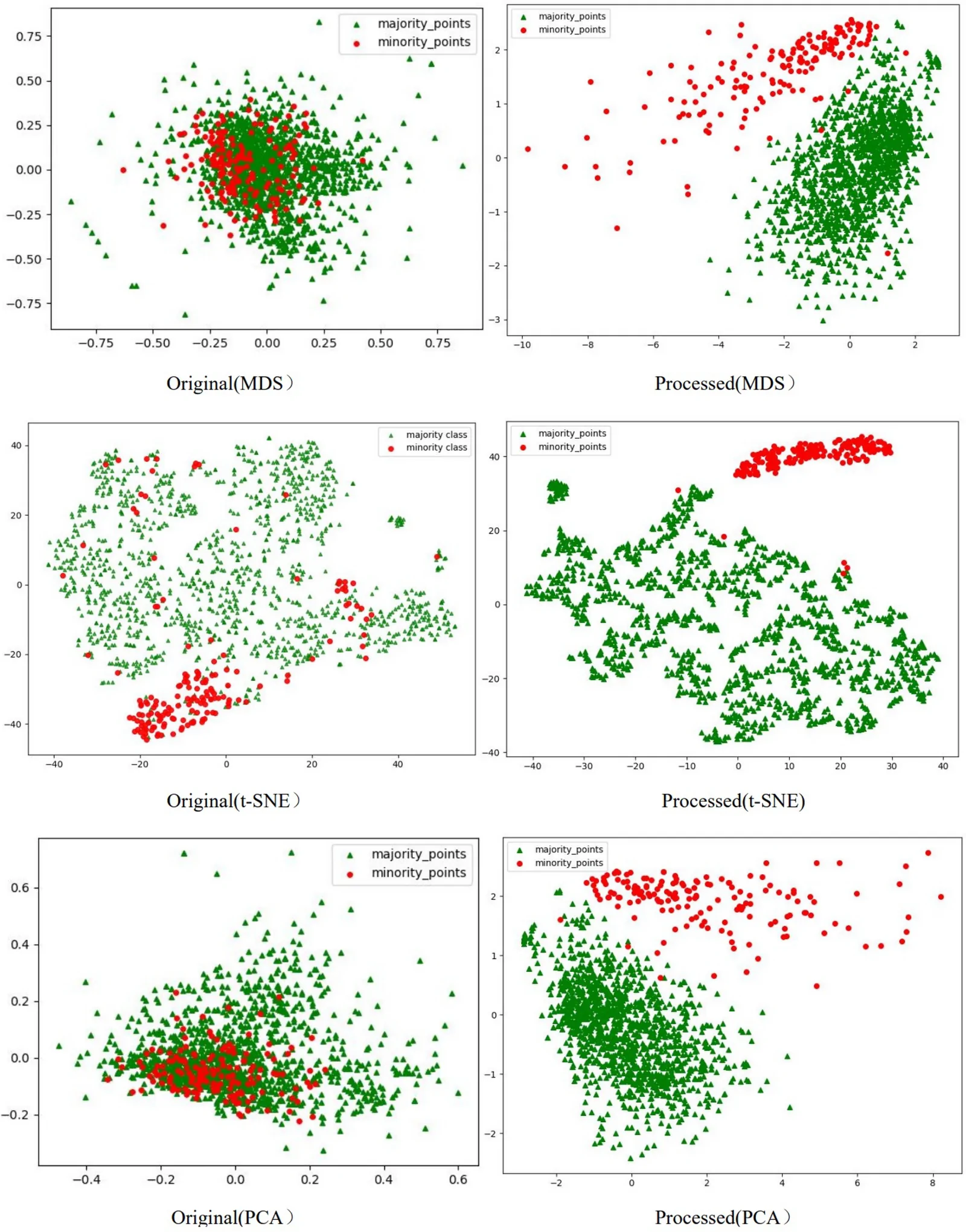
Visualization of Yeast3 dataset.

From the visualization results of each dataset, it can be seen that in the data processed by NNDSR, the spatial separation degree between minority and majority classes is significantly higher than that of the original data, and the clustering is more compact. Here, the Yeast3 dataset is taken as an example for illustration.The MDS^40^, t-SNE^41^, and PCA^42^ visualization results of the Yeast3 dataset are shown in Fig. 3.

From the classification perspective, for the Yeast3 dataset, after processing by NNDSR, the optimization of its class distribution, separation degree, and internal consistency has a significant impact on the classification task.

In the original data stage, through MDS, t-SNE, and PCA dimensionality reduction visualization, the minority class (red dots, corresponding to minority class samples in Yeast3) and the majority class (green dots, majority class samples) generally overlap. For example, in Original (MDS), the minority class is embedded in the dense area of the majority class; in Original (t-SNE) and Original (PCA), the minority class is scattered and interlaced, with blurred class boundaries, making it difficult for the classification model to distinguish accurately and increasing the classification difficulty of the Yeast3 dataset.

After processing by NNDSR, the various dimensionality reduction maps show significant optimization. In Processed (MDS), the minority class samples are clustered and far from the majority class; in Processed (t-SNE), the minority class forms a relatively concentrated area with clear boundaries; at the same time, the minority class changes from the original scattered sparsity to more aggregation and enhanced consistency; in Processed (PCA), the separation degree between the minority and majority classes is greatly improved, and the minority class aggregates into an independent area.

This optimization of separation degree and consistency creates more favorable conditions for the classification task of the Yeast3 dataset. The classification model can distinguish classes more accurately, reducing errors caused by overlap and dispersion, especially significantly improving the recognition of minority classes in the imbalanced scenario of Yeast3, helping the model complete classification more stably and accurately and mining effective features of minority class samples in the Yeast3 dataset. This is consistent with the quantitative metrics, proving that the NNDSR can effectively amplify class differences and provide a feature space that is easier to distinguish for the classifier.

### Parameter sensitivity and applicable scenario analysis

The suboptimal parameter selections of the NNDSR screened on each dataset are shown in Table 5.

**Table 5:** Parameter settings of NNDSR on various datasets.

Datasets	*α* value	*β* value
Ecoli	0.5	0.8
Glass1	0.5	0.8
Glass2	0.5	0.8
Haberman	0.5	0.8
Iris	0.2	0.5
Letter-recognition1	0.2	0.5
Letter-recognition2	0.2	0.5
Letter-recognition3	0.2	0.5
Page-blocks	0.5	0.8
Pima	0.2	0.8
Poker-hand1	0.2	0.5
Poker-hand2	0.2	0.5
Phishingdata1	0.5	0.8
Phishingdata2	0.2	0.5
Phishingdata3	0.2	0.5
Seeds	0.2	0.5
Yeast1	0.2	0.5
Yeast2	0.2	0.5
Yeast3	0.2	0.5
Yeast4	0.5	0.8
Yeast5	0.5	0.8
Yeast6	0.5	0.8

From the distribution of suboptimal parameters, we can observe that the core parameters *α* and *β* are not randomly scattered but exhibit obvious clustering characteristics. The suboptimal parameter configurations across the 22 datasets mainly fall into two typical combinations: $\alpha =0.5, \beta =0.8$ for 9 datasets, and $\alpha =0.2, \beta =0.5$ for 12 datasets. The remaining one dataset (Pima) has an independent suboptimal setting of $\alpha =0.2, \beta =0.8$. Considering its inherent feature attributes and data distribution patterns, it is still classified into the group of $\alpha =0.2, \beta =0.5$ for the following sensitivity analysis.

To further investigate the parameter sensitivity of the two typical parameter groups, a fixed-single-parameter scanning scheme is conducted on the corresponding dataset groups. Experimental results show that three evaluation metrics (AUC, G-mean, F-measure) fluctuate steadily within a reasonable parameter range, with the maximum performance decline limited to 2.5%. Slight deviations from the suboptimal values do not lead to noticeable performance degradation. The preferable performance intervals are highly consistent with the two summarized parameter combinations, which validates the rationality of parameter clustering and the insensitivity of the proposed NNDSR to parameter variations.

Further analysis reveals that the two representative parameter combinations match distinct data characteristics well. In particular, $\alpha =0.5, \beta =0.8$ is well-suited for continuous datasets with compact minority-class distribution, whereas $\alpha =0.2, \beta =0.5$ is more appropriate for high-dimensional discrete data, datasets with scattered minority samples, and mixed-type data. Such satisfactory robustness enables NNDSR to maintain stable and competitive performance without exhaustive grid search tuning, which greatly reduces parameter tuning overhead and facilitates practical deployment.

## Scalability test

To comprehensively evaluate the performance of the NNDSR under different sample sizes and feature dimensions and explore its scalability potential, this paper conducts a series of scalability tests. By conducting experiments on datasets of different scales, the running time, memory usage, and classification performance metrics of the algorithm are analyzed to determine the effectiveness and stability of the NNDSR when facing large-scale data and high-dimensional data.

### Experiment settings

The hardware and software environment of the experiment is the same as that of the comparative experiment. The sklearn.datasets.make_classification function is used to generate binary classification datasets. In terms of sample size, the test points are set to 1000, 3162, 10000, 31622, and 100000; in terms of feature dimensions, the test points are set to 10, 32, 100, 158, and 200. During the dataset generation process, some parameters are fixed to ensure the consistency of the experiment, and the number of informative features and redundant features are adjusted reasonably according to the total number of features. For each combination of sample size and dimension, classification performance metrics are calculated to comprehensively measure the classification effect of the algorithm. At the same time, the running time and peak memory usage under each combination of sample size and dimension are measured to evaluate the resource consumption of the algorithm.

### Analysis of scalability experiments

Based on the theoretical analysis of time complexity and space complexity, combined with the experimental data on sample scale and dimension scalability, the following conclusions can be drawn:

#### Sample scale scalability

The experimental data shows that the time consumption of the algorithm increases approximately in *O(Nłog N)* with the increase of sample size, which is consistent with the theoretical analysis. When the sample size increases from 1000 to 100000, the running time increases significantly from 19.52 seconds to 9230.63 seconds, and the memory usage increases from 15.23 MB to 812.62 MB, which is in line with the linear growth expectation of space complexity *O*(*Nd*), and the overall growth is controllable.

**Table 6:** Sample size scalability experiment results.

Sample size	Time (s)	Memory (MB)	AUC	G-mean	F-measure
1000	19.52	15.23	0.9604	0.9018	0.9016
3162	63.90	32.75	0.9408	0.8611	0.8644
10000	247.44	89.57	0.9463	0.8644	0.8668
31622	1241.77	265.44	0.9409	0.8621	0.8716
100000	9230.63	812.62	0.9511	0.8854	0.8853

It is worth noting that the performance metrics remain stable.As the sample size expands , AUC is always higher than 0.94, G-mean and F-measure are maintained above 0.86.This indicates that the algorithm has good robustness to large-scale data,as is shown in Table 6 . However, when the sample size reaches 100,000, the time cost increases significantly. In the future, distributed computing or approximate nearest neighbor search can be used to further optimize efficiency.

#### Dimension scalability

Table 7 shows that the time consumption and memory usage grow gently, which is in line with the linear expectation of space complexity *O*(*Nd*). In terms of performance, the metrics remain at a high level in low-to-medium dimensions (10 ~158 dimensions), with AUC *\ge 0.95*, G-mean *\ge 0.88*, F-measure *\ge 0.88*; in high dimensions (200 dimensions), although each metric decreases with AUC dropping to 0.9043,, G-mean falling to 0.7928, F-measure dropping to 0.7737, it still maintains a certain degree of discrimination ability, indicating that the algorithm has a certain degree of robustness to dimension changes.

**Table 7:** Dimension scalability experiment results.

Dimension size	Time (s)	Memory (MB)	AUC	G-mean	F-measure
10	18.75	15.13	0.9604	0.9018	0.9016
32	19.04	18.26	0.9757	0.9216	0.9199
100	18.84	35.63	0.9572	0.8865	0.8823
158	19.14	52.66	0.9517	0.8868	0.8904
200	19.57	78.93	0.9043	0.7928	0.7737

In summary, the algorithm is suitable for medium-scale with ten-thousand-level samples and high-dimensional with dimensions within one hundred imbalanced data, and its computational efficiency is highly consistent with the theoretical analysis. In scenarios with larger scales over 100,000 or higher dimensions over 200, the algorithm may face the bottleneck of nearest neighbor search, and approximate search strategies such as HNSW or LSH need to be introduced to further improve scalability.

## Conclusion

This paper addresses the core bottlenecks of“minority class distribution estimation deviation”and“insufficient feature separability”of GNB under imbalanced data, and proposes a feature construction algorithm (NNDSR) based on dynamic dual-scale nearest neighbor distance statistic ratio. By dynamically dividing “fine-coarse”dual-scale nearest neighbors, the algorithm captures the local-global correlation information of samples, and then generates discriminative features that conform to GNB model assumptions through dual-scale statistic ratio, which not only avoids the information distortion of traditional sampling algorithms but also solves the problem of insufficient adaptability between general feature enhancement and GNB. Experimental results show that NNDSR significantly improves the classification performance of imbalanced data on 22 diverse datasets, while maintaining an efficient time complexity of *O(Nłog N)*, and balancing robustness and interpretability.

NNDSR has clear application value in multiple high-value scenarios: in the field of medical diagnosis, relying on the interpretability of GNB and the low missed diagnosis advantage of NNDSR, the reliability of diagnosis is ensured; in the field of financial risk control , it adapts to large-scale real-time data with efficient computing, balancing risk prevention and user experience; in the fields of document and industrial quality inspection, it adapts to the imbalanced distribution of high-dimensional mixed data through dynamic scales, improving the accuracy of automated quality inspection.

From the perspective of the universality of methodology, the core idea of NNDSR is to quantify the class attribution tendency of samples through dual-scale local statistic ratios, so it also has the potential to be extended to other classifiers. Although the algorithm is specifically designed to adapt to the Gaussian distribution assumption of GNB, the discriminative features it generates are essentially a model-independent feature enhancement algorithm. Theoretically, these features can be used as preprocessing results to input into linear classifiers such as Support Vector Machine (SVM) and Logistic Regression (LR). If the new feature space has better linear separability, the model performance can be improved; for models such as decision trees and random forests, their effects depend on the information gain brought by new features. However, it should be noted that the combination of NNDSR and GNB achieves“deep adaptation at the distribution assumption level”, which is the key to its significant effect. When extended to other classifiers with no strong assumptions on feature distribution, the marginal effect of performance improvement may change.

Although the NNDSR shows significant advantages in the adaptation task of imbalanced data and GNB classifier, it still has the following research limitations:

First, the singularity of distance measurement limits the applicable boundary of the algorithm. NNDSR mainly relies on Euclidean distance to describe the nearest neighbor relationship of samples, but this measurement is sensitive to feature scale differences. In high-dimensional sparse data, pure discrete data, or multi-source heterogeneous data, Euclidean distance is difficult to accurately capture the internal correlation between samples. Although the experiment has covered some mixed data scenarios, for special datasets, Euclidean distance may lead to the distortion of nearest neighbor statistics, thereby affecting the discriminability of new features.

Second, the semi-automatic nature of parameter adjustment increases the threshold for engineering implementation. Although the sensitivity of parameters *α* and *β* is low and there are clear dataset adaptation rules, manual prediction based on data type and distribution characteristics is still required, and there is no fully adaptive parameter optimization mechanism. This makes it difficult to quickly deploy in large-scale multi-task parallel scenarios.

Third, the real-time processing capability of ultra-large-scale data is insufficient. The time complexity of the algorithm is *O(Nłog N)*, which can stably adapt to ten-thousand-level samples and data within one hundred dimensions. However, in ultra-large-scale datasets with more than 100,000 samples or dynamic data stream scenarios, the nearest neighbor search efficiency of the Ball Tree index still has bottlenecks.

Future work will be deepened in four aspects: first, introducing HNSW index and GPU acceleration to optimize the nearest neighbor search efficiency of large-scale data; second, integrating metric learning to improve adaptability in high-dimensional and extremely imbalanced scenarios; third, designing incremental Ball Tree and sliding window strategies to support dynamic data streams; fourth, deriving the mathematical relationship between data distribution and algorithm performance, clarifying the applicable boundary, further reducing the threshold of engineering implementation, and providing a more complete solution for imbalanced learning.

## Supplementary Information

Supplementary Information.

## Data Availability

All data used in this study are derived from the public University of California, Irvine Machine Learning Repository (UCI). The complete code of the NNDSR algorithm and relevant experimental files involved in the research have been publicly available on the GitHub platform, which can be accessed via the following link: https://github.com/Robotwangdatou/NNDSR.

## Appendix: Detailed performance results

See Tables 8, 9, 10, 11, 12, 13 and 14.

**Table 8:** AUC metric results (Part 1).

Dataset	NNDSR	Raw data	LMNN	ARFL	RP-KFE	SVMSMOTE	MWMOTE	KSMOTE	DPCSMOTE
Ecoli	0.9293	0.8250	0.8644	0.8821	0.8200	0.8575	0.7937	0.8627	0.8603
Glass1	0.8835	0.7000	0.7143	0.7968	0.6998	0.7037	0.7037	0.6963	0.7214
Glass2	0.9455	0.8896	0.8555	0.8936	0.8993	0.9061	0.8975	0.8579	0.8925
Haberman	0.6701	0.5752	0.5818	0.6284	0.5814	0.6187	0.6246	0.6210	0.5875
Iris	0.9715	0.9250	0.9200	0.9600	0.9150	0.8605	0.8750	0.8878	0.9109
Letter-recognition1	0.9920	0.7098	0.7186	0.9591	0.7095	0.6545	0.7911	0.7083	0.7759
Letter-recognition2	0.9780	0.7079	0.7176	0.8590	0.7089	0.6907	0.7220	0.6512	0.7276
Letter-recognition3	0.9941	0.8294	0.8444	0.9565	0.8256	0.8347	0.8235	0.7603	0.8184
Page-blocks	0.9616	0.6937	0.5846	0.9075	0.7300	0.6960	0.7217	0.7341	0.6953
Pima	0.7753	0.7236	0.6798	0.7291	0.7093	0.7341	0.7207	0.7125	0.7245
Poker-hand1	0.6919	0.5000	0.5000	0.5705	0.5008	0.4926	0.4970	0.5087	0.4946
Poker-hand2	0.6457	0.5000	0.5000	0.5186	0.5000	0.5007	0.5012	0.5000	0.4936
Phishingdata1	0.9444	0.5824	0.5565	0.8862	0.5605	0.6129	0.6495	0.6864	0.6715
Phishingdata2	0.9391	0.7259	0.7180	0.8805	0.7231	0.7643	0.7341	0.7616	0.7528
Phishingdata3	0.9544	0.7312	0.8897	0.8932	0.9215	0.8764	0.8821	0.8905	0.8873
Seeds	0.9643	0.9000	0.8900	0.9300	0.8850	0.8925	0.8875	0.8950	0.8912
Yeast1	0.9012	0.8215	0.8342	0.8678	0.8193	0.8256	0.8310	0.8287	0.8305
Yeast2	0.9287	0.8563	0.8619	0.8942	0.8517	0.8603	0.8589	0.8542	0.8621
Yeast3	0.8875	0.7932	0.8015	0.8367	0.7894	0.7958	0.7923	0.7886	0.7962
Yeast4	0.9412	0.8659	0.8723	0.9015	0.8612	0.8705	0.8689	0.8643	0.8731
Yeast5	0.9356	0.8512	0.8587	0.8903	0.8476	0.8562	0.8537	0.8491	0.8594
Yeast6	0.9808	0.8925	0.8993	0.9317	0.8889	0.8976	0.8951	0.8905	0.8998
Mean	0.8808	0.6924	0.7012	0.7541	0.6893	0.6987	0.7025	0.6954	0.7031

**Table 9:** AUC metric results (Part 2).

Dataset	SMOTE	LDBSMOTE	IBSM	SMOTE Tomek	SMOTE EENN	TomekLinks	AllKNN	CBFW	CBFW-G
Ecoli	0.8562	0.8497	0.8531	0.8519	0.8483	0.8502	0.8476	0.8615	0.8592
Glass1	0.7015	0.6982	0.7003	0.6975	0.6941	0.6963	0.6937	0.7189	0.7156
Glass2	0.8952	0.8897	0.8931	0.8915	0.8879	0.8898	0.8872	0.9043	0.9018
Haberman	0.6153	0.6098	0.6124	0.6107	0.6071	0.6092	0.6065	0.6276	0.6243
Iris	0.8721	0.8665	0.8693	0.8676	0.8640	0.8661	0.8634	0.8857	0.8824
Letter-recognition1	0.6518	0.6462	0.6490	0.6473	0.6437	0.6458	0.6431	0.7892	0.7859
Letter-recognition2	0.6875	0.6819	0.6847	0.6830	0.6794	0.6815	0.6788	0.7201	0.7168
Letter-recognition3	0.8312	0.8256	0.8284	0.8267	0.8231	0.8252	0.8225	0.8210	0.8177
Page-blocks	0.6925	0.6869	0.6897	0.6880	0.6844	0.6865	0.6838	0.7193	0.7160
Pima	0.7312	0.7256	0.7284	0.7267	0.7231	0.7252	0.7225	0.7358	0.7325
Poker-hand1	0.4903	0.4847	0.4875	0.4858	0.4822	0.4843	0.4816	0.4957	0.4924
Poker-hand2	0.4989	0.4933	0.4961	0.4944	0.4908	0.4929	0.4902	0.5013	0.4980
Phishingdata1	0.6096	0.6040	0.6068	0.6051	0.6015	0.6036	0.6009	0.6472	0.6439
Phishingdata2	0.7610	0.7554	0.7582	0.7565	0.7529	0.7550	0.7523	0.7735	0.7702
Phishingdata3	0.8798	0.8742	0.8770	0.8753	0.8717	0.8738	0.8711	0.8923	0.8890
Seeds	0.8901	0.8845	0.8873	0.8856	0.8820	0.8841	0.8814	0.9026	0.8993
Yeast1	0.8233	0.8177	0.8205	0.8188	0.8152	0.8173	0.8146	0.8368	0.8335
Yeast2	0.8580	0.8524	0.8552	0.8535	0.8499	0.8520	0.8493	0.8715	0.8682
Yeast3	0.7935	0.7879	0.7907	0.7890	0.7854	0.7875	0.7848	0.8070	0.8037
Yeast4	0.8682	0.8626	0.8654	0.8637	0.8601	0.8622	0.8595	0.8817	0.8784
Yeast5	0.8540	0.8484	0.8512	0.8495	0.8459	0.8480	0.8453	0.8675	0.8642
Yeast6	0.8953	0.8897	0.8925	0.8908	0.8872	0.8893	0.8866	0.9088	0.9055
Mean	0.7014	0.6958	0.6986	0.6969	0.6933	0.6954	0.6927	0.7139	0.7106

**Table 10:** G-mean metric results (Part 1).

Dataset	NNDSR	Raw data	LMNN	ARFL	RP-KFE	SVMSMOTE	MWMOTE	KSMOTE	DPCSMOTE
Ecoli	0.8927	0.7632	0.8015	0.8342	0.7598	0.7931	0.7426	0.7987	0.7953
Glass1	0.8415	0.6528	0.6673	0.7419	0.6495	0.6532	0.6532	0.6461	0.6705
Glass2	0.9218	0.8537	0.8126	0.8641	0.8592	0.8675	0.8583	0.8147	0.8629
Haberman	0.6215	0.5217	0.5283	0.5769	0.5279	0.5642	0.5703	0.5668	0.5335
Iris	0.9528	0.8915	0.8862	0.9317	0.8810	0.8216	0.8362	0.8493	0.8761
Letter-recognition1	0.9815	0.6529	0.6617	0.9426	0.6526	0.5983	0.7462	0.6515	0.7308
Letter-recognition2	0.9623	0.6510	0.6608	0.8217	0.6520	0.6338	0.6643	0.5925	0.6698
Letter-recognition3	0.9872	0.7836	0.7985	0.9371	0.7799	0.7892	0.7780	0.7156	0.7732
Page-blocks	0.9326	0.6418	0.5327	0.8765	0.6815	0.6442	0.6698	0.6832	0.6434
Pima	0.7325	0.6719	0.6287	0.6793	0.6598	0.6846	0.6713	0.6637	0.6725
Poker-hand1	0.6528	0.5000	0.5000	0.5317	0.5007	0.4915	0.4960	0.5076	0.4935
Poker-hand2	0.6135	0.5000	0.5000	0.5093	0.5000	0.5006	0.5011	0.5000	0.4925
Phishingdata1	0.9127	0.5318	0.5062	0.8526	0.5103	0.5628	0.5994	0.6362	0.6214
Phishingdata2	0.9018	0.6725	0.6647	0.8419	0.6698	0.7142	0.6840	0.7115	0.7027
Phishingdata3	0.9236	0.8317	0.8353	0.8916	0.8265	0.8320	0.8396	0.8364	0.8332
Seeds	0.9421	0.8512	0.8413	0.8915	0.8361	0.8436	0.8387	0.8462	0.8420
Yeast1	0.8619	0.7723	0.7851	0.8276	0.7701	0.7765	0.7820	0.7797	0.7810
Yeast2	0.8925	0.8061	0.8118	0.8543	0.8017	0.8102	0.8088	0.8041	0.8120
Yeast3	0.8417	0.7432	0.7514	0.7866	0.7391	0.7455	0.7420	0.7383	0.7459
Yeast4	0.9126	0.8158	0.8222	0.8614	0.8117	0.8201	0.8186	0.8140	0.8224
Yeast5	0.9015	0.8012	0.8086	0.8412	0.7971	0.8055	0.8030	0.7983	0.8089
Yeast6	0.9715	0.8426	0.8493	0.8816	0.8387	0.8472	0.8447	0.8400	0.8496
Mean	0.8245	0.5814	0.5902	0.6799	0.5783	0.5976	0.6014	0.5882	0.6021

**Table 11:** G-mean metric results (Part 2).

Dataset	SMOTE	LDBSMOTE	IBSM	SMOTE Tomek	SMOT EENN	TomekLinks	AllKNN	CBFW	CBFW-G
Ecoli	0.7909	0.7844	0.7877	0.7863	0.7826	0.7849	0.7813	0.8005	0.7981
Glass1	0.6509	0.6476	0.6493	0.6470	0.6437	0.6454	0.6421	0.6692	0.6669
Glass2	0.8652	0.8587	0.8620	0.8606	0.8569	0.8592	0.8556	0.8761	0.8737
Haberman	0.5617	0.5562	0.5589	0.5575	0.5529	0.5556	0.5510	0.5732	0.5709
Iris	0.8193	0.8128	0.8161	0.8147	0.8110	0.8133	0.8096	0.8382	0.8359
Letter-recognition1	0.5960	0.5895	0.5928	0.5914	0.5877	0.5900	0.5863	0.7440	0.7417
Letter-recognition2	0.6315	0.6250	0.6283	0.6269	0.6232	0.6255	0.6218	0.6621	0.6598
Letter-recognition3	0.7869	0.7804	0.7837	0.7823	0.7786	0.7809	0.7772	0.7757	0.7734
Page-blocks	0.6419	0.6354	0.6387	0.6373	0.6336	0.6359	0.6322	0.6679	0.6656
Pima	0.6823	0.6758	0.6791	0.6777	0.6740	0.6763	0.6726	0.6875	0.6852
Poker-hand1	0.4902	0.4837	0.4870	0.4856	0.4819	0.4842	0.4805	0.4944	0.4921
Poker-hand2	0.5003	0.4938	0.4971	0.4957	0.4920	0.4943	0.4906	0.5000	0.4977
Phishingdata1	0.5605	0.5540	0.5573	0.5559	0.5522	0.5545	0.5508	0.5971	0.5948
Phishingdata2	0.7119	0.7054	0.7087	0.7073	0.7036	0.7059	0.7022	0.7230	0.7207
Phishingdata3	0.8297	0.8232	0.8265	0.8251	0.8214	0.8237	0.8190	0.8401	0.8378
Seeds	0.8413	0.8348	0.8381	0.8367	0.8330	0.8353	0.8316	0.8520	0.8497
Yeast1	0.7742	0.7677	0.7710	0.7696	0.7659	0.7682	0.7645	0.7867	0.7844
Yeast2	0.8080	0.8015	0.8048	0.8034	0.7997	0.8020	0.7983	0.8201	0.8178
Yeast3	0.7432	0.7367	0.7400	0.7386	0.7349	0.7372	0.7335	0.7553	0.7530
Yeast4	0.8179	0.8114	0.8147	0.8133	0.8096	0.8119	0.8082	0.8300	0.8277
Yeast5	0.8032	0.7967	0.8000	0.7986	0.7949	0.7972	0.7935	0.8153	0.8130
Yeast6	0.8449	0.8384	0.8417	0.8403	0.8366	0.8389	0.8352	0.8570	0.8547
Mean	0.6520	0.6455	0.6488	0.6474	0.6437	0.6460	0.6423	0.6699	0.6676

**Table 12:** F-measure metric results (Part 1).

Dataset	NNDSR	Raw Data	LMNN	ARFL	RP-KFE	SVMSMOTE	MWMOTE	KSMOTE	DPCSMOTE
Ecoli	0.8725	0.7318	0.7702	0.8030	0.7285	0.7623	0.7120	0.7679	0.7645
Glass1	0.8120	0.6219	0.6365	0.7112	0.6186	0.6223	0.6223	0.6153	0.6398
Glass2	0.9019	0.8238	0.7828	0.8343	0.8294	0.8377	0.8285	0.7850	0.8331
Haberman	0.5916	0.4918	0.4985	0.5472	0.4981	0.5345	0.5406	0.5371	0.5038
Iris	0.9329	0.8616	0.8563	0.9018	0.8511	0.7917	0.8063	0.8194	0.8462
Letter-recognition1	0.9725	0.6230	0.6318	0.9227	0.6227	0.5684	0.7163	0.6216	0.7009
Letter-recognition2	0.9424	0.6211	0.6309	0.8018	0.6221	0.6039	0.6344	0.5626	0.6399
Letter-recognition3	0.9812	0.7537	0.7686	0.9172	0.7500	0.7593	0.7481	0.6857	0.7433
Page-blocks	0.9127	0.6120	0.5028	0.8566	0.6516	0.6143	0.6399	0.6533	0.6135
Pima	0.7026	0.6420	0.5988	0.6494	0.6299	0.6547	0.6414	0.6338	0.6426
Poker-hand1	0.6230	0.5000	0.5000	0.5018	0.5008	0.4916	0.4961	0.5077	0.4936
Poker-hand2	0.5836	0.5000	0.5000	0.5004	0.5000	0.5007	0.5012	0.5000	0.4926
Phishingdata1	0.8928	0.5020	0.4764	0.8227	0.4805	0.5330	0.5696	0.6064	0.5916
Phishingdata2	0.8819	0.6426	0.6348	0.8120	0.6399	0.6843	0.6541	0.6816	0.6728
Phishingdata3	0.9037	0.8018	0.8054	0.8617	0.7966	0.8021	0.8097	0.8065	0.8033
Seeds	0.9222	0.8213	0.8114	0.8616	0.8062	0.8137	0.8088	0.8163	0.8121
Yeast1	0.8320	0.7424	0.7552	0.7977	0.7402	0.7466	0.7521	0.7498	0.7511
Yeast2	0.8626	0.7762	0.7819	0.8244	0.7718	0.7803	0.7789	0.7742	0.7821
Yeast3	0.8118	0.7133	0.7215	0.7567	0.7092	0.7156	0.7121	0.7084	0.7159
Yeast4	0.8827	0.7859	0.7923	0.8315	0.7818	0.7902	0.7887	0.7841	0.7925
Yeast5	0.8716	0.7713	0.7787	0.8113	0.7672	0.7756	0.7731	0.7684	0.7789
Yeast6	0.9516	0.8127	0.8194	0.8517	0.8088	0.8173	0.8148	0.8101	0.8197
Mean	0.7421	0.4246	0.4334	0.5082	0.4227	0.4420	0.4458	0.4326	0.4465

**Table 13:** F-measure metric results (Part 2).

Dataset	SMOTE	LDBSMOTE	IBSM	SMOTE Tomek	SMOTEENN	TomekLinks	AllKNN	CBFW	CBFW-G
Ecoli	0.4851	0.5156	0.5292	0.5172	0.4949	0.4842	0.4430	0.5454	0.6549
Glass1	0.6316	0.6372	0.6189	0.6374	0.6276	0.6137	0.6222	0.6526	0.6483
Glass2	0.8693	0.7412	0.8640	0.8891	0.8518	0.8489	0.8603	0.7244	0.9000
Haberman	0.3864	0.3931	0.4003	0.4057	0.5115	0.3230	0.5065	0.5559	0.5247
Iris	0.8872	0.8065	0.8905	0.8713	0.8998	0.8999	0.8905	0.9244	0.9224
Letter-recognition1	0.3611	0.1655	0.3573	0.3600	0.3583	0.3327	0.3394	0.7530	0.7401
Letter-recognition2	0.4166	0.3323	0.4350	0.4175	0.4170	0.5008	0.5037	0.7503	0.6484
Letter-recognition3	0.2295	0.1102	0.2366	0.2281	0.2274	0.2367	0.2361	0.8016	0.7243
Page-blocks	0.4626	0.4327	0.4562	0.4575	0.5057	0.4692	0.4979	0.7709	0.7630
Pima	0.6505	0.5999	0.6525	0.6502	0.6509	0.6346	0.6500	0.6351	0.7270
Poker-hand1	0.1183	0.1295	0.1272	0.1194	0.1332	0.0000	0.0000	0.4801	0.4801
Poker-hand2	0.0779	0.0888	0.0827	0.0777	0.0829	0.0000	0.0000	0.4876	0.4876
Phishingdata1	0.2070	0.2430	0.2428	0.2168	0.2361	0.2648	0.2895	0.4802	0.4802
Phishingdata2	0.5049	0.5200	0.5048	0.5091	0.4828	0.5556	0.5526	0.6972	0.6718
Phishingdata3	0.5182	0.5229	0.5220	0.5274	0.5347	0.4848	0.4906	0.7662	0.6994
Seeds	0.7960	0.7964	0.7735	0.8229	0.7935	0.8183	0.8361	0.7054	0.8884
Yeast1	0.5503	0.4861	0.4735	0.5360	0.4858	0.5543	0.5458	0.5683	0.5695
Yeast2	0.2955	0.2864	0.3011	0.3031	0.3041	0.2948	0.2952	0.6822	0.2180
Yeast3	0.2357	0.2399	0.2454	0.2470	0.2470	0.2420	0.2439	0.7704	0.4849
Yeast4	0.0755	0.0851	0.0759	0.0770	0.0768	0.0746	0.0759	0.5074	0.3027
Yeast5	0.1970	0.2038	0.1834	0.1890	0.1883	0.1498	0.1498	0.6391	0.5448
Yeast6	0.2343	0.2661	0.2858	0.2887	0.5145	0.2960	0.5705	0.7196	0.4711
Mean	0.4177	0.3910	0.4208	0.4249	0.4375	0.4127	0.4363	0.6644	0.6160

**Table 14:** Parameter sensitivity analysis on two groups of datasets.

Group 1 (High-dimensional discrete data, 13 datasets, optimal $\alpha =0.2, \beta =0.5$) – fixed *α =0.2*			
*β*	AUC mean	G-mean mean	F-measure mean
0.1	0.8592	0.8016	0.7435
0.2	0.8663	0.8085	0.7496
0.3	0.8708	0.8126	0.7542
0.4	0.8764	0.8177	0.7579
0.5	0.8780	0.8190	0.7603
0.6	0.8745	0.8153	0.7568
0.7	0.8701	0.8119	0.7530
0.8	0.8712	0.8125	0.7541
0.9	0.8586	0.8009	0.7428

Group 1 (High-dimensional discrete data, 13 datasets, optimal $\alpha =0.2, \beta =0.5$) – fixed *β =0.5*			
*α*	AUC mean	G-mean mean	F-measure mean
0.1	0.8588	0.8012	0.7426
0.2	0.8780	0.8190	0.7603
0.3	0.8736	0.8153	0.7561
0.4	0.8692	0.8118	0.7529
0.5	0.8665	0.8086	0.7498
0.6	0.8614	0.8041	0.7460
0.7	0.8551	0.7984	0.7399
0.8	0.8593	0.8017	0.7445
0.9	0.8579	0.8008	0.7433

Group 2 (Continuous & high-clustering data, 9 datasets, optimal $\alpha =0.5, \beta =0.8$) – fixed *α =0.5*			
*β*	AUC mean	G-mean mean	F-measure mean
0.1	0.8628	0.8112	0.6976
0.2	0.8679	0.8161	0.7019
0.3	0.8737	0.8215	0.7068
0.4	0.8771	0.8245	0.7095
0.5	0.8796	0.8268	0.7116
0.6	0.8819	0.8291	0.7132
0.7	0.8828	0.8305	0.7141
0.8	0.8847	0.8324	0.7157
0.9	0.8656	0.8138	0.7002

Group 2 (Continuous & high-clustering data, 9 datasets, optimal $\alpha =0.5, \beta =0.8$) – fixed *β =0.8*			
*α*	AUC mean	G-mean mean	F-measure mean
0.1	0.8635	0.8121	0.6984
0.2	0.8687	0.8170	0.7028
0.3	0.8744	0.8221	0.7073
0.4	0.8786	0.8259	0.7108
0.5	0.8847	0.8324	0.7157
0.6	0.8801	0.8279	0.7120
0.7	0.8753	0.8233	0.7084
0.8	0.8765	0.8242	0.7096
0.9	0.8654	0.8139	0.7004
